# Biological Responses to Diesel Exhaust Particles (DEPs) Depend on the Physicochemical Properties of the DEPs

**DOI:** 10.1371/journal.pone.0026749

**Published:** 2011-10-21

**Authors:** Eun-Jung Park, Jinkyu Roh, Min-Sung Kang, Soo Nam Kim, Younghun Kim, Sangdun Choi

**Affiliations:** 1 Department of Molecular Science and Technology, Ajou University, Suwon, Korea; 2 Department of Chemical Engineering, Kwangwoon University, Seoul, Korea; 3 Inhalation Toxicology Center, Korea Institute of Toxicology, Jeongeup, Korea; Tulane University, United States of America

## Abstract

Diesel exhaust particles (DEPs) are the main components of ambient particulate materials, including polyaromatic hydrocarbons (PAHs), n-PAHs, heavy metals, and gaseous materials. Many epidemiological, clinical, and toxicological studies have shown that ambient particles, including DEPs, are associated with respiratory disorders, such as asthma, allergic rhinitis, and lung cancer. However, the relationship between the biological response to DEPs and their chemical composition remains unclear. In this study, we investigated the physicochemical properties of DEPs before toxicological studies, and then administered a single intratracheal instillation of DEPs to mice. The mice were then killed 1, 7, 14 and 28 days after DEP exposure to observe the biological responses induced by DEPs over time. Our findings suggest that DEPs engulfed into cells induced a Th2-type inflammatory response followed by DNA damage, whereas DEPs not engulfed into cells induced a Th1-type inflammatory response. Further, the physicochemical properties, including surface charge, particle size, and chemical composition, of DEPs play a crucial role in determining the biological responses to DEPs. Consequently, we suggest that the biological response to DEPs depend on cell-particle interaction and the physicochemical properties of the particles.

## Introduction

Ambient particles are known as both initiators and enhancers of the clinical manifestations of both allergic and non-allergic airway disease in industrialized countries, and diesel exhaust particles (DEPs) are one of main components of ambient particles. DEP exposure can induce acute irritation of the eyes and throat, light-headedness, and nausea. Further, they have been associated with the worsening of respiratory symptoms, such as cough, phlegm, chronic bronchitis, and asthma. Epidemiologic studies also suggested a strong link between DEP exposure and detrimental health concerns, including cardiopulmonary morbidity and mortality [1], [2], [3].

It has been established that DEPs are known to generate reactive oxygen species (ROS) on intracellular uptake, and ROS generation is attributed to the chemical composition of the particles, such as transition metals and organic chemicals. ROS generated by DEP exposure can also lead to oxidative stress, which in turn triggers a variety of cellular consequences, such as DNA damage, apoptosis, inflammatory responses, and antioxidant defense activation/depletion [4], [5], [6], [7], [8].

The incidence of allergic airway disease has increased in parallel with the increasing use of fossil fuels. Data collected until 2009 shows that asthma is a problem worldwide, affecting an estimated 300 million individuals (Global Initiative for Asthma, GINA). DEPs act deeply in the nasal epithelium by directing cytokine gene expression toward a Th2 profile, enhancing local antigen-specific immunoglobulin (Ig) E production and driving in vivo isotype switch to IgE production [9]. Additionally, DEPs interfere with not only the maturation but also the function of dendritic cells, thus suggesting that DEPs play a role in Th2-type immune deviations [10]. Lungs of mice repeatedly exposed to DEPs plus ovalbumin (OVA) showed higher expression of major histocompatibility complex (MHC) class II cells and cells expressing CD11c, DEC205, CD80, CD86, F4/80, and CD19 than those of mice exposed to the vehicle, DEPs, or OVA. In addition, splenic mononuclear cells primed by DEPs plus OVA produced a greater amount of interleukin (IL)-4, IL-5, and IL-13 after in vitro antigen stimulation than those primed by vehicle, DEPs, or OVA [11]. DEPs also significantly suppressed mRNA expression and protein production of interferon (IFN)-γ, but did not affect those of IL-4 and IL-5 [12]. In addition, polyaromatic hydrocarbons (PAHs) have been extracted from DEPs, and DEPs enhanced B-cell differentiation both *in vitro* and *in vivo* [13]. PAHs from roadside emission also significantly enhanced cytokine secretion (IL-4 and IL-8) and histamine release from purified basophils [14].

Furthermore, several studies have indicated that DEP exposure is associated with oxidative damage to DNA, and this might be associated with an increased risk of cancer [4], [15], [16], [17]. In a previous study, DEP exposure was shown to downregulate the expression of murine double minute 2 (Mdm2) protein, a negative regulator of p53, and upregulate the expression of Bax, a pro-apoptotic protein and endogenous target of p53-dependent transcriptional activation [18]. Additionally, exposure of human airway epithelial cells to DEPs caused either the up- or downregulation of 197 of 313 detectable miRNAs (62.9%) by at least 1.5-fold. Molecular network analysis of the putative targets of the 12 most-altered miRNAs indicated that DEPs exposure is associated with inflammatory response pathways and a strong tumorigenic disease signature [19], [20]. Human–hamster hybrid cells exposed to DEPs also exhibited a dose-dependent increase in the mutation yield at the CD59 locus, with minimal cytotoxicity [20].

To date, the relationship between the physicochemical properties of DEPs and the biological response triggered by exposure to DEPs remains unclear, despite ample evidence on the adverse health effects of DEPs. In this study, we first investigated the physicochemical properties of DEPs before proceeding with toxicological studies; for the latter, mice were administered DEPs via a single intratracheal instillation, and the biological responses on days 1, 7, 14, and 28 were evaluated by postmortem examination.

## Materials and Methods

### Animal and housing conditions

Five-week-old ICR mice were purchased from Orient Bio Inc. (Gyeonggi-do, Korea) and were acclimated to room conditions for 2 weeks before the initiation of the study. The environmental conditions were controlled at a constant temperature of 23±3°C, relative humidity of 55±10%, and 12-h light/dark cycle with light of intensity 150–300 lx, and ventilation 10–20 times/h. Three animals were housed in each suspended, stainless-steel wire cage (255×465×200 mm^3^) during the acclimation, pretreatment, and treatment periods. Each animal was identified by a tail tattoo and a cage card. Gamma-ray-irradiated standard laboratory rodent pellet diet (PMI Nutrition International, Richmond, IN, USA) and municipal tap water sterilized by ultraviolet light were provided to the animals *ad libitum*. All experiments were performed in accordance with the guidelines and regulations of the Korea Institute of Toxicology and were approved by the Institutional Animal Care and Use Committee (IACUC) (Approval Number: BJ09060). All animal facilities in this study were accredited by the Association for Assessment and Accreditation of Laboratory Animal Care International (AAALAC).

### DEP analysis

Morphological properties of DEP were observed by a high-resolution transmission electron microscope (HRTEM; JEM-3010, JOEL, Japan) and an electrophoretic light-scattering spectroscope (ELS; ELS-8000, Otsuka electronics, Japan). The chemical composition of DEPs was ascertained by the analysis of an energy-dispersive X-ray spectroscope (EDS; Oxford instruments, England) and a fourier transform infrared (FT-IR) spectroscope (Nicolet 6700, Thermo scientific, USA).

### Intratracheal instillation and sample preparation

To study the time course of response to DEP exposure, mice were treated with DEPs or phosphate buffered saline (PBS). Intratracheal instillation was performed by a qualified technician from the Korea Institute of Toxicology, one of the GLP institutes in Korea, and mice were administered DEPs using a 24-gauge catheter at a dose of 100 µg/kg under light anesthesia induced by tiletamine. The animals were killed 1, 7, 14, or 28 days after exposure. The control group comprised 4 mice treated with PBS.

The body weights of the control and DEP-treated mice were measured before exposure and before necropsy on days 1, 7, 14, and 28 after treatment. At the selected time intervals, 1.2-ml blood samples were collected from the saphenous vein of each mouse after treatment. The blood samples harvested from the four mice were pooled to make 1 test sample, yielding 4 test samples separately from sixteen mice for further analysis for the cell count, cell phenotype, cytokine concentration, and histamine concentration (n = 4). Whole blood was centrifuged at 3,000 rpm for 10 min to prepare serum, and 500–600 µL of serum was obtained from each mouse. Bronchoalveolar lavage (BAL) fluid was obtained by cannulating the trachea and lavaging the lungs with 1 ml of cold sterile PBS (0.15 M, pH 7.2) free of Ca^2+^ and Mg^2+^; approximately 500–600 µL of BAL fluid was harvested per mouse, centrifuged at 3,000 rpm for 10 min, and analyzed for cell count and cytokine concentration.

### BAL fluid analysis

Total cells in the BAL fluid were quantified by hemocytometric counting, with cell differentials performed on cytocentrifuged preparations fixed in methanol and stained with Diff-Quick (Thermo Shandon, PA, USA). Distributions of the alveolar macrophages, neutrophils, and lymphocytes were assessed by their characteristic cell shapes.

### Measurement of cytokine concentrations

The concentrations of each cytokine in the supernatant of the BAL fluid and serum were determined using commercially available enzyme-linked immunosorbent assay (ELISA) kits (eBioscience, San Diego, CA, USA) according to the manufacturer's recommendations. Briefly, each well of a microplate was coated with 100 µL of the capture antibody and incubated overnight at 4°C. After washing and blocking the cells with assay diluent and BAL fluid, serum or standard antibody was added to individual wells; the plates were then maintained at room temperature for 2 h. The plates were washed, and biotin-conjugated detecting antibody was added to each well and incubated at room temperature for 1 h. The plates were washed again and further incubated with avidin-horseradish peroxidase for 30 min before antibody detection by using 3,3′,5,5′-tetramethylbenzidine solution. Finally, reactions were stopped by adding 1 M H_3_PO_4_, and the absorbance at 450 nm was measured with an ELISA reader (Molecular Devices, Sunnyvale, CA, USA). The amount of cytokine was calculated from the linear portion of the generated standard curve [21], [22].

### Immunophenotyping

All monoclonal antibodies were purchased from eBioscience (San Diego, CA, USA). T cells (CD3, 1∶50), B cells (CD19, 1∶50), natural killer (NK) cells (DX5, 1∶100), CD4+ T cells(CD4+, 1∶160), and CD8+ T cells (CD8+, 1∶50) were identified using directly conjugated anti-mouse antibodies. Briefly, blood was blocked with Fc-block (eBioscience, San Diego, CA, USA) to reduce non-specific antibody binding. Cells were then incubated in the dark with 10 µL of the appropriate fluorochrome-conjugated antibody for 20 min at 4°C. Cells were then washed with fluorescence-activated cell sorter (FACS) buffer. The blood was lysed for 5 min with FACS lysis buffer (BD Bioscience, Franklin Lakes, NJ, USA) at room temperature and then re-washed with FACS buffer. Finally, each sample was fixed with 1% paraformaldehyde until further analysis. Flow cytometry analysis was performed using the FACSCalibur system (BD Biosciences, Franklin Lakes, NJ, USA). Control samples were matched for each fluorochrome. Data were analyzed using CellQuest software (Becton Dickinson, Franklin Lakes, NJ, USA) [21], [22].

### Measurement of histamine concentration

Histamine concentration was measured using a mouse histamine ELISA kit (Wuhan EIAab Science Co., LTD., Wuhan, China) according to the manufacturer's protocol. Briefly, after adding 100 µL of standard, blank, or sample per well, the plates were incubated for 2 h at 37°C. The liquid was removed from each well; detection reagent A working solution was added to each well; and the plate was incubated at 37°C for 1 h. After washing, detection reagent B working solution was added to each well, and the plate was incubated at 37°C for 1 h. After re-washing, the substrate solution was added to each well and incubated at 37°C for 15 min, without exposure to light. Finally, the reaction was terminated by the addition of stop solution, and the absorbance was measured at 450 nm by using a microplate reader (Molecular Devices, Sunnyvale, CA, USA). The amounts of secreted collagen were calculated from the linear portion of the prepared standard curve.

### Protein expression in tissue

Lung tissue was homogenized with a protein extraction solution (PRO-PREP™, Cat. No. 17081, iNtRON biotechnology, Kyunggi, Korea), and lysates were centrifuged at 13,000 rpm for 10 min. The protein concentration was measured by the Bradford method (Bio-Rad Protein Assay, Bio-Rad Laboratories Inc, Hercules, CA), and equal amounts of proteins (40 µg) were separated on a SDS/1%-polyacrylamide gel, and then transferred to a nitrocellulose membrane (Hybond ECL, Amersham Pharmacia Biotech Inc., Piscataway, NJ, USA). Blots were blocked for 2 h at room temperature with 5% (w/v) non-fat dried milk in Tris-buffered saline [10 mM Tris (pH 8.0) and 150 mM NaCl] solution containing 0.05% Tween-20. The membranes were immunoblotted with the following primary specific antibodies: rabbit polyclonal antibodies to COX-2 (1∶500 dilution, Cayman Chemical, MI, USA) and phospho-p53 (1∶1000 dilution, Cell Signaling Technology, Inc. Beverly, MA, USA); rabbit monoclonal antibody to β-actin (1∶2000 dilution, Cell Signaling Technology, Inc. Beverly, MA, USA); mouse monoclonal antibodies to p-STAT3, p-IκBα (both, 1∶200 dilution, Santa Cruz Biotechnology Inc. CA, USA), mesothelin, p53 (both, 1∶1000 dilution, Santa Cruz Biotechnology Inc. CA, USA), iNOS (1∶1000 dilution, BD Biosciences, CA, USA), and RANTES (1∶4 dilution, eBioscience, San Diego, CA, USA); and goat polyclonal to TGFβ1 (1∶1000 dilution, Santa Cruz Biotechnology Inc. CA, USA). The blot was then incubated with the corresponding conjugated anti-mouse, anti-rabbit, and anti-goat IgG-horseradish peroxidase (1∶2000 dilution, Santa Cruz Biotechnology Inc.). Immunoreactive proteins were detected using the electrochemiluminescence Western blotting detection system.

### Statistical analysis

DEP-treated groups were compared to those obtained from the control group. The values were compared using Dunnett's *t*-test after one-way ANOVA, and the levels of significance were determined by comparison with the control group.

## Results

### Properties of DEPs

**Figure 1:**
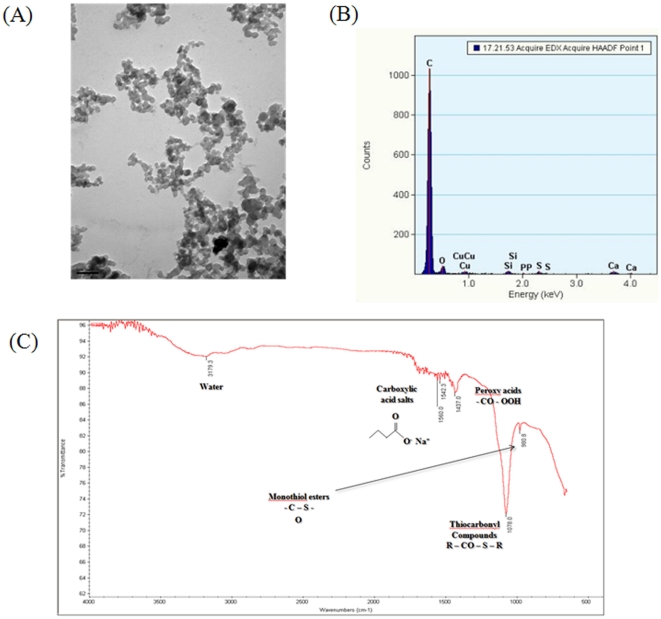
Physicochemical properties of DEPs suspended in PBS. (A) TEM image, (B) Energy-dispersive X-ray spectroscopy, and (C) FT-IR image.

The DEPs investigated in this study were found to be slightly aggregated particles of diameter 1 µm, comprising small particles of diameter 20 nm (Fig. 1A); the DEPs were a mass of metal oxide compounds, which are generally formed when diesel is burnt (Fig. 1B). DEPs were found to have a surface charge of −39.1±0.8 mV and a hydrophilic surface because of the presence of oxides (Fig. 1C).

### Change in body weight by DEPs

**Figure 2:**
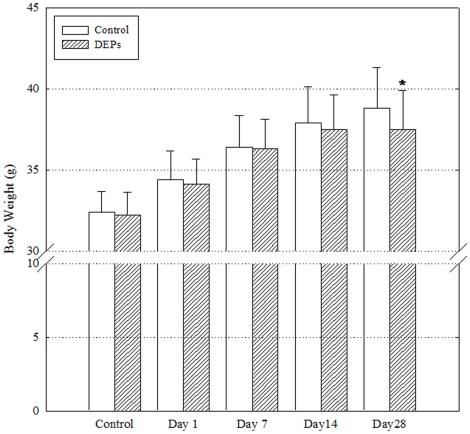
Changes in body weight after a single instillation of DEPs. Body weight of mice (n = 16) in the control and day 28 groups before exposure and on days 1, 7, 14, and 28 after exposure. *P<0.05.

Before exposure to DEPs, the mean body weight in the control and DEP-treated groups were 32.4±1.3 g and 32.4±1.4 g, respectively (Fig 2). On day 28 after treatment, the mean body weight in the DEP-treated group (37.5±2.38 g) was significantly lesser than that in the control group (38.8±2.48 g).

### BAL fluid composition

**Figure 3:**
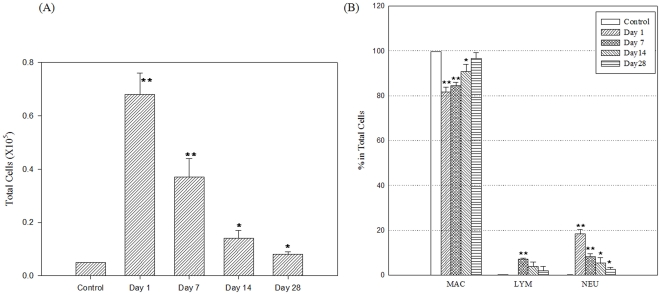
Changes in the cell distribution in BAL fluid after instillation of DEPs (n = 4). Mice were administered a single intratracheal instillation of DEPs at a dose of 10 mg/kg and then killed on the designated day (1, 7, 14, or 28 days after exposure). The cells in the BAL fluid were quantified by hemocytometric counting (A), and the distributions of alveolar macrophages, neutrophils, and lymphocytes were assessed on the basis of their characteristic cell shapes (B). The cell number in each group was expressed as mean ± SD. *; P<0.05, **; P<0.01.

The total number of cells in the BAL fluid was significantly greater in the DEP-treated mice than in the controls: the value in the control group was (0.05±0.00)×10^5^ cells, while that in the DEP-treated mice examined on days 1, 7, 14, and 28 after treatment were (0.68±0.08), (0.37±0.07), (0.14±0.03), and (0.08±0.01)×10^5^ cells, respectively (Fig. 3A). The percentage of macrophages in the BAL fluid decreased rapidly on day 1 (81.7±2.1%), and continued to decrease over time (Fig. 3B). At all the defined time points, the percentage of neutrophils in the BAL fluid samples of DEP-treated mice was significantly greater than that in the controls: in controls, it was 0.1±0.1% and in DEP-treated mice examined on days 1, 7, 14, and 28 after treatment, it was 18.3±2.1%, 8.3±1.2%, 5.4±2.4%, and 2.4±1.0%, respectively. On day 7, the percentage of lymphocytes was significantly greater in the DEP-treated mice (7.13±0.51%) than in the controls.

### Cytokines in BAL fluid

As shown in Table 1, the concentrations of the proinflammatory cytokines, IL-1, TNF-α, and IL-6, were significantly greater in the DEP-treated mice than in the controls at all the time points; in particular, the DEP-treated mice exhibited an obvious increase in the concentration of TNF-α from day 1 to day 14 after treatment. Similarly, the IL-6 concentration in the BAL fluid of DEP-treated mice was 2.7-fold that in the control mice on day 1. In addition, the levels of IL-12 peaked on day 7 and were greater than that in the control by about 3.3-, 10.4-, 8.6-, and 1.4-fold on days 1, 7, 14, and 28 after treatment, respectively. The concentration of IFN-γ was 2.5-fold that in the control on day 1 after treatment. At all time points, the concentrations of the Th2-type cytokines, IL-4, IL-5, IL-10, and IL-13 as well as IL-17 were also significantly greater in the DEP-treated mice than those in the controls, but the increase in the concentration of these cytokines were lower than that of the Th1-type cytokines.

**Table 1:** Changes in the cytokine levels in BAL fluid after a single instillation of DEPs (n = 4).

	Control	Day 1	Day 7	Day 14	Day 28
IL-1	11.86±0.92	19.84±3.29*	20.48±4.34*	16.93±1.63*	17.28±2.09*
TNF-α	13.25±0.86	54.00±4.43**	33.24±5.57**	42.18±5.37**	19.89±1.48*
IL-6	42.71±1.40	115.98±10.73**	76.92±4.91**	58.43±1.59*	56.95±4.22*
IL-2	19.55±0.70	26.52±1.91*	29.13±5.39*	27.07±0.93*	24.92±4.53
IL-12	11.18±0.82	37.38±7.81*	116.56±32.82*	96.40±21.36*	16.15±1.79*
IFN-γ	13.31±0.46	33.42±4.64**	19.91±3.47*	18.98±1.14*	16.00±1.31
IL-4	22.24±3.57	27.39±4.59	26.16±3.10	25.07±1.35	29.83±3.17
IL-5	32.11±1.95	36.41±4.03	49.79±2.02*	34.69±3.01	36.70±3.54
IL-10	54.70±1.53	95.72±6.93**	97.26±6.51**	80.88±4.86*	103.20±6.85**
IL-13	26.92±4.50	33.18±8.33*	56.01±7.74*	48.76±7.08*	38.65±3.54*
IL-17	75.63±0.96	106.94±4.49*	127.70±5.83**	117.40±3.45**	119.17±4.29**

Note: BAL fluid was harvested at each time point and pooled (500 µL per mouse) to 4 test samples per group for further analysis. The level in each group was calculated as mean ± SD of the values measured.

*P<0.05;

**P<0.01.

### Phagocytosis of DEPs by cells in BAL fluid

**Figure 4:**
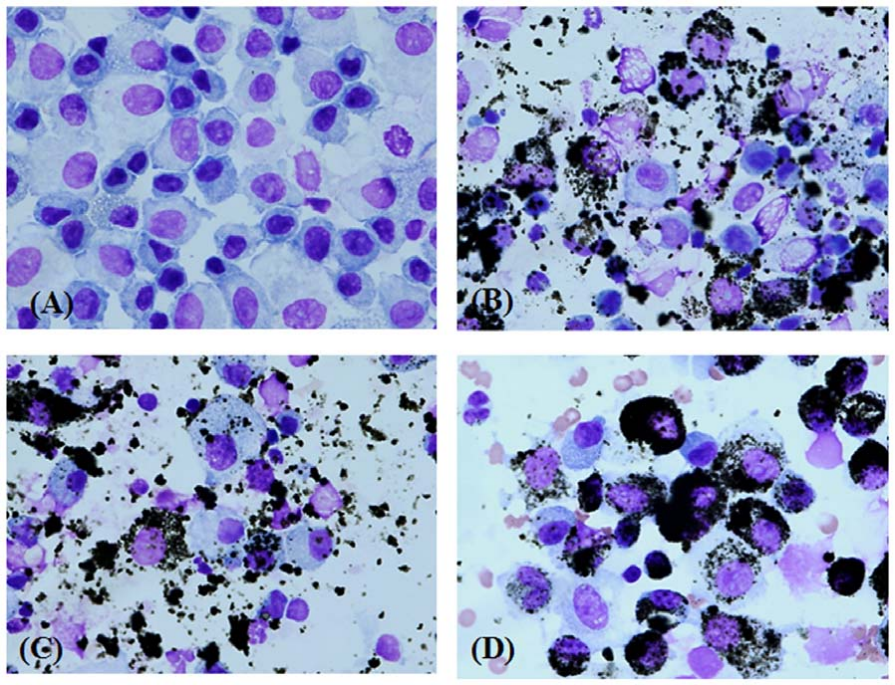
Images of cells in BAL fluid after a single instillation of DEPs. Mice were administered a single intratracheal instillation of DEPs at a dose of 10 mg/kg, and BAL fluid samples were harvested on the designated days: (A) control, (B) 7 days, (C) 14 days, and (D) 28 days after exposure. The results were confirmed from all the mice used, and representative images are shown.

Figure 4 shows an image of cells in the BAL fluid harvested at each time point. As shown in Figure 3, DEPs were suspended in BAL fluid until day 14 after treatment and were partially distributed in the cytoplasm of cells. Phagocytosis by immune cells in BAL fluid was completed on day 28 after treatment, which was indicated by the mainly cytoplasmic and partially nuclear distribution.

### Protein expression in lung tissue

**Figure 5:**
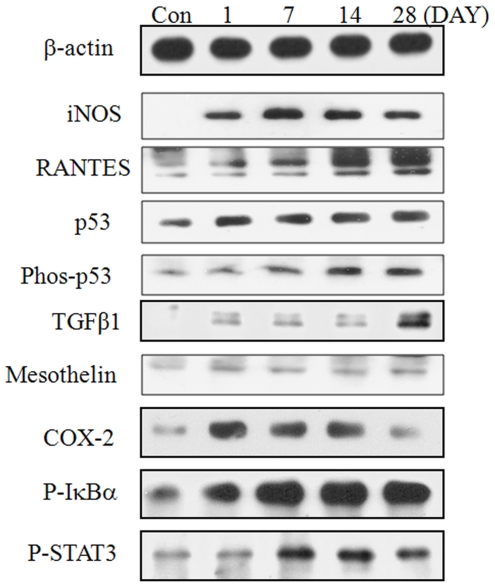
Changes in the protein expression in lung tissue after a single instillation of DEPs. Results were confirmed by several separate experiments, and representative images are shown.

Protein expression of RANTES, p53, and phospho-p53 in lung tissue increased in a time-dependent manner and that of TGF-β was clearly induced only on day 28 after treatment (Fig. 5). The concentration of inducible nitric oxide synthatase (iNOS) increased significantly during the experimental period and peaked on day 7 after treatment, whereas expression of COX-2 was upregulated from day 1 to day 14 after treatment. The effect of DEP exposure on the expression of mesothelin protein was minimal. The protein expression levels of p-IκBα, p-STAT3, and NFκB p65 peaked on day 14 after treatment.

### Cytokines in the blood

As shown in Table 2, the levels of the proinflammatory cytokines, IL-1, TNF-α, and IL-6 were significantly greater than those in the controls at all time points; the levels peaked on day 7 after treatment and were about 4.1-, 2.1-, and 12.8-fold, respectively. The levels of IL-12 peaked on day 7 (2.1-fold that in the controls) after treatment. The levels of IL-4 and IL-5 also peaked on day 7 after treatment and were 3.6- and 5.2-fold, respectively. The levels of IL-2 and IL-17 remained almost the same throughput the experimental period.

**Table 2:** Changes in the cytokine levels in the blood after a single instillation of DEPs (n = 4).

	Control	Day 1	Day 7	Day 14	Day 28
IL-1	4.30±0.03	8.63±0.07**	17.60±0.50**	10.30±0.12**	7.70±0.07**
TNF-α	3.92±0.04	6.78±0.07**	8.28±0.13**	7.45±0.02**	6.78±0.11*
IL-6	4.21±0.11	10.94±0.12**	53.79±8.32**	24.60±0.19**	8.75±0.28**
IL-2	ND	1.83±0.01	0.97±0.00	0.97±0.00	0.75±0.00
IL-12	57.54±1.76	77.74±7.41*	117.43±28.83*	69.11±13.62*	92.08±18.29*
IFN-γ	ND	ND	ND	ND	ND
IL-4	1.14±0.02	2.85±0.02**	4.07±0.06**	3.34±0.03**	2.03±0.01*
IL-5	3.00±0.02	5.15±0.02*	15.45±0.36**	4.29±0.02*	4.29±0.01*
IL-10	ND	ND	ND	ND	ND
IL-13	ND	ND	ND	ND	ND
IL-17	25.17±2.24	30.49±0.70*	30.56±1.82*	29.48±1.64	28.82±1.10

*Note.* Serum was harvested on each time point and pooled (500 µL per mouse) to 4 test samples per group for further analysis. The level in each group was calculated as the mean ± SD of the values measured.

*P<0.05;

**P<0.01.

### Secretion of TGF-β and histamine

**Figure 6:**
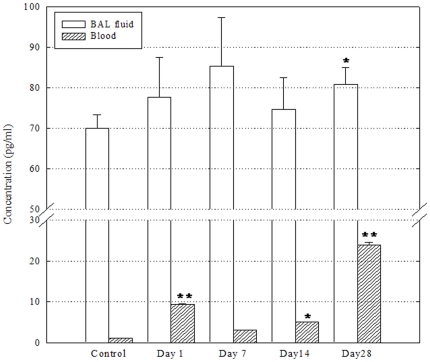
Levels of TGF-β in BAL fluid and blood after a single instillation of DEPs (n = 4). BAL fluid and serum samples were harvested and pooled on days 1, 7, 14, and 28 after DEP instillation, and pooled (500 µL per mouse) to 4 test samples per group for further analysis, respectively. The levels in each group were calculated as mean ± SD of the values measured. *P<0.05; **P<0.01.

TGF-β levels in blood were significantly greater than that in the controls at all time points, whereas the levels of TGF-β in BAL fluid were significantly greater than those in the controls only on day 28 after treatment (Fig. 6). Blood levels of TGF-β were 9.35±0.14, 3.05±0.05, 5.08±0.06, and 23.81±0.76 pg/ml on days 1, 7, 14, and 28 after treatment, respectively, while that in the controls was 1.12±0.00 pg/ml.

**Figure 7:**
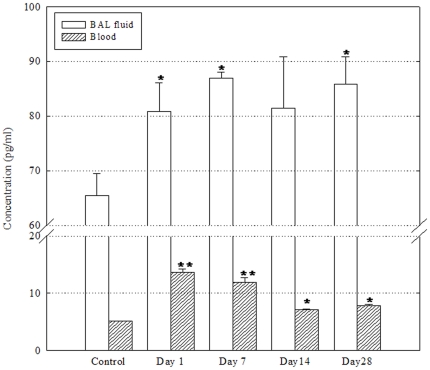
Levels of histamine in BAL fluid and in blood following a single instillation with DEPs (n = 4). BAL fluid and serum were harvested and pooled on day 1, 7, 14, and 28 after DEP instillation. The level in each group was expressed as the mean ± SD of the values measured. *P<0.05; **P<0.01.

The post-treatment levels of histamine in the BAL fluid and blood were significantly greater than that in the controls (Fig. 7). The level of histamine in the BAL fluid was almost the same as that in the control throughout the experimental period (1.2- to 1.3-fold that in the control). The blood levels of histamine were 13.7±0.5, 11.9±0.8, 7.2±0.1, and 7.8±0.2 pg/ml on days 1, 7, 14, and 28 after treatment, while the level in the controls was 5.1±0.02 pg/ml.

### Cell composition of lymphocytes

**Figure 8:**
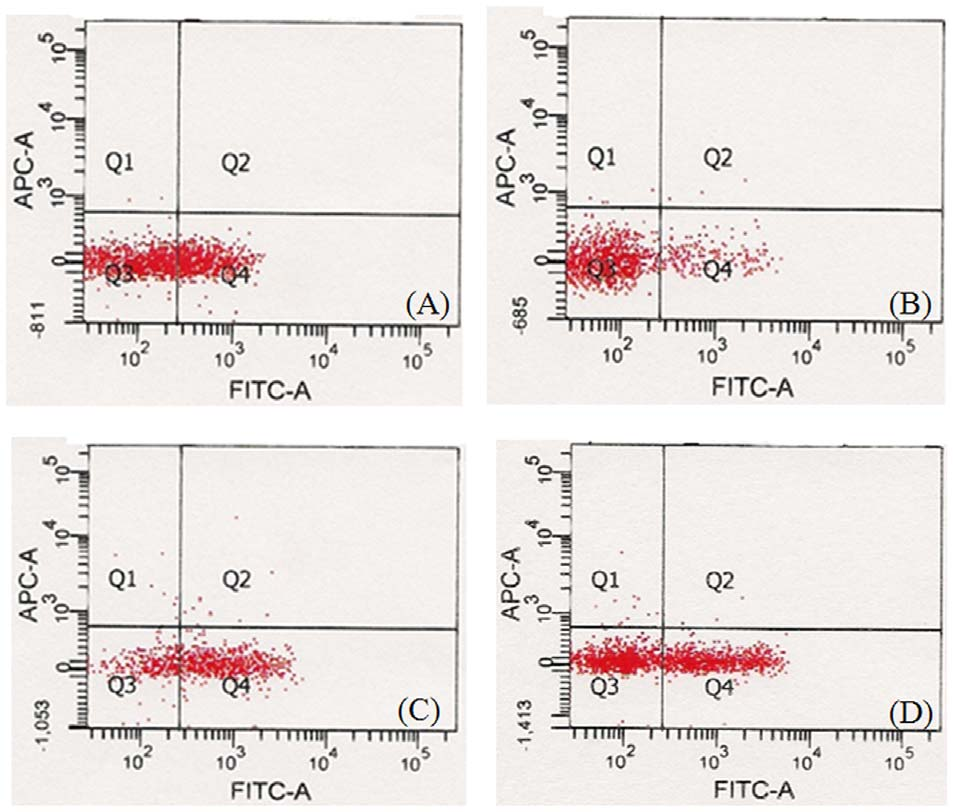
The levels of cell phenotype in blood following a single instillation with DEPs (n = 4). Whole blood was harvested from 16 mice on day 1, 7, 14, and 28 after DEP instillation, and pooled to make 4 samples for cell phenotype analysis. (A) control, (B) day 1, (C) day 7, (D) day 14.

As shown in Table 3 and Figure 8, the cell distribution of lymphocytes shifted to a T-cell-dominant direction with the increase in CD8+ T cells on day 1, but shifted to a B-cell-dominant direction with the recovery of the ratio of CD4+/CD8+ on day 7 after treatment. On day 14, the ratio of CD4+/CD8+ cells were clearly increased, implying an increase in the number of CD4+ T cells; however, the distribution of T and B cells did not change significantly. Furthermore, a significant increase in the number of NK cells was shown during the experimental period.

**Table 3:** Lymphocyte phenotypes in blood after DEPs instillation (n = 4).

	Control	DAY 1	DAY 7	DAY 14	DAY 28
NK	0.1±0.1	0.4±0.1*	0.6±0.2*	0.7±0.1**	0.7±0.4*
NKT	0.0±0.0	0.2±0.0*	0.6±0.6	0.2±0.1	0.3±0.2
T	61.2±3.4	80.9±7.0*	41.0±8.9*	54.8±5.6	58.3±5.8
B	38.7±3.5	18.6±7.0*	57.7±8.1*	44.3±5.6	40.6±6.1
CD4+/CD8+	4.3±0.1	2.7±0.2*	4.0±0.5	5.6±0.5*	4.5±0.5

Note: The level in each group is the mean ± SD of value was measured at each time point.

*; P<0.05,

**; P<0.01.

## Discussion

Exposure to DEPs is an environmental and occupational health concern, and epidemiological, clinical and toxicological data indicate that exposure to DEPs induces both hypersensitive disease and cancer. Considering the fact that hypersensitive disease and cancer involve different immune responses, we investigated the relationship between the physicochemical properties of DEPs and the biological responses induced by DEP exposure.

DEPs are composed of a carbonaceous core with adsorbed organic compounds, sulfates, and trace elements. Soluble organic compounds, including PAHs, can represent up to 60% of the mass of the particle. The main targets of DEPs introduced into the body via the bronchial tubes are immune cells in the BAL fluid and airway epithelial cells. The human epithelium contains several types of polarized cells with specific functions. The permeability of a molecule depends mainly on its electric charge of the molecule and to a lesser extent on its molar mass [23]. Therefore, neutrally charged, small particles may pass the cell membrane more easily than charged, large ones. In our previous study on PM2.5, the particle size in PBS was 909.9±318.4 nm, with a surface charge/conductance of −3.71 mV/30500 µS and an average mobility of −0.29 (µ/s)/(V/cm); the particles were composed of toxic heavy metals, such as Cr, As, Cd, and Pb, as well as other ionic components. PM2.5 instilled via the trachea was easily engulfed by the immune cells in the BAL fluid on day 1 after treatment and consistently induced a Th1-type inflammatory response, along with a slight increase in subG1 phase cells and an increase in the expression of CD8+ T cells (CD4+ T cell on day 1 after treatment). In this study, the surface charge of DEPs in PBS was −38.37 mV and the heavy metals on the surface were relatively non-toxic. In addition, the DEPs were not completely engulfed by the immune cells in the BAL fluid until day 14 after treatment and induced a confused immune response during the experimental period. The total cells in BAL fluid increased at all times tested, indicating that DEPs did not induce apoptosis or necrosis.

The rapid increase in the burden of hypersensitive disease has undeniably occurred in parallel with rapid industrialization and urbanization in many parts of the world. Several studies have shown that DEPs act as adjuvants to allergens and hence increase the sensitization response [2], [3], [4], [24]. Furthermore, Ma and Ma (2002) reported that the organic component of DEPs may skew the immunity toward a Th2 response, whereas the particulate component of DEPs may stimulate both the Th1 and Th2 responses. In this study, DEP exposure was found to cause an obvious increase in the TNF-α levels in the BAL fluid to 4.1-fold that in the control, with a marked increase in CD8+ T-cell distribution on day 1 after treatment, but induced B-cell dominance with rapid recovery of the ratio of CD4+/CD8+ T cells on day 7. In addition, on day 7, the concentrations of IL-1, IL-6, and IL-5 in the blood were 4.1-, 12.8-, and 5.2-fold that in the control, respectively, whereas the IL-12 concentration in the BAL fluid increased to 10.4-fold that in the control. Considering the fact that the cellular response in the blood stream was caused by exposure to DEPs engulfed by immune cells, we suggest that the changes observed from day 1, i.e., Th1-type inflammatory response and CD8+ T-cell dominance, may have been effected by the surface charge and surface chemicals, and those from day 7, i.e., Th2-type inflammatory response, may be attributed to the release of the soluble chemical components of DEPs engulfed into immune cells. Our suggestions are supported by the rapid decrease in the levels of IL-12 and the rapid increase in the levels of IL-10 and IL-17 in the BAL fluid after complete phagocytosis of DEPs by immune cells in the BAL fluid on day 28. However, there is a possibility that other cells in the lung including epithelial cells will also release some cytokines and measurement in BAL fluid cannot differentiate them from immune cells. Furthermore, the cell response will also change as time goes on. Some cells including immune cells and parenchymal cells will undergo apoptosis and necrosis. These may change the cell composition in the lung, thereby resulting in the release of cytokines.

Ma and Ma (2002) also reported that both the organic and particulate components of DEPs exhibit different biological actions, but both induce cellular oxidative stress. Together, these effects exacerbate respiratory allergy and induce DNA damage, eventually leading to the development of lung tumors. Further, the oxidative stress mediated by DEPs may be induced via various mechanisms, such as the direct generation of ROS from the surface of particles; release of soluble compounds, such as transition metals or organic compounds; altered function of mitochondria or NADPH-oxidase, and activation of inflammatory cells capable of generating ROS and reactive nitrogen species [25], [26]. Furthermore, the increase in the mutations induced by DEP was significantly reduced by concurrent treatment with phagocytosis inhibitors [20]. In this study, expression of transcriptional proteins, such as p-IkBα, pSTAT3, and p65, peaked on day 14, whereas the expression of p53, phospho-p53, RANTES, and TGF-β in lung tissue peaked on day 28, after a slight time-dependent increase after DEP exposure. One of the major functions of TGF-β is the inhibition of growth through the blockage of the cell cycle in the late-G1 phase and inflammatory resolution by immune suppression. The most fundamental function of p53 is to serve as an essential growth checkpoint for protecting cells against cellular transformation, thereby resulting in blockage of the cell cycle [27], [28], [29], [30]. The phospho–p53 complex is the active form of p53, and RANTES has chemotactic activity for monocytes/macrophages, T cells, eosinophils, and basophils [31]. In addition, the expression of iNOS increased clearly until day 28 after DEP exposure, whereas that of cyclooxygenase-2 (COX-2) increased until day 14 after DEP exposure. COX-2 is the inducible isoform of COX, which is a key enzyme in the conversion of arachidonic acid to prostaglandins and other eicosanoids. iNOS is the inducible isoform of NOS, which is the enzyme that catalyzes the formation of nitric oxide, a regulator of vascular permeability. Both COX-2 and iNOS are inducible by oxidative stress, and iNOS is inducible by COX-2. p53 also downregulates the angiogenic process at various levels [32], [33].

Furthermore, in this study, all cytokines, except TNF-α and IL-12, were measured in the BAL fluid; in particular, the levels of Th2-type cytokines showed a slight increase after DEP exposure until day 14. DEPs were not completely engulfed by immune cells in the BAL fluid until day 14. However, DEPs were absorbed into the blood stream, thereby inducing Th2-type inflammatory responses on day 7 after DEP exposure. The expression of p53, phospho-p53, and TGF-β in lung tissue peaked on day 28, by which time DEPs had been completely engulfed by the immune cells in the BAL fluid. On day 14, a B-cell-dominant trend was absent, despite phagocytosis of DEPs by the immune cells in the BAL fluid and transference of the particles into the blood stream. The ratio of CD4+/CD8+ T cells showed a trend towards greater concentration of CD4+ T cell since day 1, despite the consistent and significant post-treatment increase in the distribution of NK cells expressing markers of CD8+ T cells. In addition, the levels of TGF-β increased again in both BAL and blood, whereas the level of histamine was increased again in the BAL fluid only from day 14 onwards. On the basis of the results of this study, we also suggest that DEPs engulfed by immune and epithelial cells may induce an early hypersensitive response and subsequent DNA damage triggered by the breaking down and release of the soluble chemical components of DEPs in cells.
